# Chicago Hospital for Women and Children

**Published:** 1879-06

**Authors:** L. Anna Ballard


### Clinical Reports.

### ARTICLE VI.

### CHICAGO HOSPITAL FOR WOMEN AND CHILDREN.

SERVICE OF DR. MARY H. THOMPSON. (Reported by L. Anna Ballard, M. D.)

The following cases of ovariotomy, taken from the records of the Chicago Hospital for Women and Children, present some points of interest, as these three successive cases terminated in perfect recovery, and illustrate the value of Lister's antiseptic method of operating.

*Case I.*—J. C. Aged 31. Single. Born in Scotland. Occupation, general housework.

She has generally been in good health, but has never been a strong woman. First menstruated at the age of 16. Never suffered unusual pain at menstrual periods. In May, 1877, she was kicked in the iliac region, which left a " soreness for several weeks."

In January, 1878, she first noticed a tumor in the right ovarian region just before a menstrual period, after which it enlarged rapidly. She suffered no inconvenience until July following, when sharp, stinging pains were felt in the left side. Menstruation occurred regularly up to the date of operation.

She consulted Dr. R. G. Bogue, who advised her to come to this hospital. Entered August 5, 1878. On examination by Drs. Bogue and Thompson, fluctuation was revealed by palpation. The enlargement of abdomen was symmetrical; depth of uterus over ten Ctm.; breathing oppressed. It was decided to perform paracentesis by means of trocar and canula inserted into the linea alba. Dr. Bogue drew from the cyst 8 litres of light brown fluid. This was done to confirm the diagnosis.

Aug. 15th. The patient was again examined by Dr. Bogue, who found the cyst filling with fluid, and advised her to leave the hospital until time for operation, which he thought would be in about two months.

Oct. 10th. She was readmitted. Abdomen full and tense. Breathing oppressed. Intense pain in the back, from which cause she had been for two weeks unable to sleep in a recumbent posture. Bowels inactive. Kidneys not secreting well.

She was put upon tonics and diuretics until the 19th, when Dr. Bogue, assisted by Dr. Thompson and others, removed a multilocular cyst weighing, with its contents, 15 kilog. There were adhesions all around the cyst. The cyst wall, though handled with due care, was so tender as to rupture, and let a large portion of the thick, tenacious fluid flow into the abdominal cavity, which was thoroughly sponged out with warm carbolized water; this process requiring several minutes.

The tumor was in the right broad ligament, involving ovary and Fallopian tube. A double ligature of white silk braid (carbolized) was applied to the pedicle very near the uterus, tied in two parts, and cut near the knots. The pedicle was then cut about one Ctm. from the ligature and dropped back into the abdomen. There was no hæmorrhage. Drainage tubes were inserted in the lower end of the incision, and the wound closed by four hare-lip pins, including the peritoneum in the sutures, and by five intermediate superficial silk sutures.

The operation was performed under the carbolized spray of 5 per cent. strength, sponges and instruments were kept well carbolized, and Lister's dressings were afterward applied.

The recovery was rapid and uninterrupted. On Nov. 14th the patient walked three miles. She has on several occasions since reported herself well.

*Case II.*—Mrs. M. F. C. Widow. Aged 50. Born in New York. Mother of five children. Menstruation began when the patient was about 14 years old, and recurred regularly to the time of the first pregnancy. For the past 24 years she has been subject to dysentery, having several attacks yearly. Sixteen years since was injured in a riot, the frontal bone being fractured. From this wound 9 small pieces of bone were removed. A few months afterward she began having "fits," which continued for ten years.

She first noticed abdominal enlargement 7 years ago. It increased very slowly. She was treated in New York city for ascites. Three years since she was attacked by menorrhagia, which was followed by a sickness of which she can give little account. Once after this sickness she had an excessive menstrual flow, and since that time she has had complete prolapsus of the uterus.

She first entered this hospital Oct. 29th, 1877. Measured at umbillicus 108 Ctm. Depth of uterus 14 Ctm. Kidneys not secreting well. Was treated with tonics and diuretics without decrease of abdominal measurement.

Nov. 22d, 1877. Dr. Thompson called the hospital staff in consultation. The enlargement was diagnosticated to be, one or more ovarian cysts. Dr. Thompson aspirated the tumor, drawing away 12½ kilo. of a thick, foaming, brown fluid. The tonic and diuretic treatment was continued. During the winter and spring the patient was troubled with dysentery and various other temporary complaints.

January 25th, 1878. As respiration was becoming oppressed, and the patient could not make up her mind to submit to an operation, the tumor was again aspirated. The fluid which was taken away measured 12½ kilo., and was of a dark brown color.

August 26th, 1878. She left the hospital, intending to return in the fall for an operation.

November 19th, 1878. The patient returned. Her general health was good. Abdomen very large. Respiration not greatly disturbed.

December 4th, Dr. Mary H. Thompson, assisted by Drs. Byford, Bogue and others, removed a multilocular cyst weighing 1⅓ myrg. pounds and containing 13 kilo. of dark, ropy fluid. There were no adhesions about the superior portion, but at the base of the tumor the adhesions were extensive and strong. This cyst was taken from the left, broad ligament; the pedicle was ligated with a double, white silk carbolized braid which divided the pedicle into two parts, and was tied on opposite sides. The ligatures were then cut short, and the pedicle, cut about one

Ctm. from the ligatures, was dropped into the abdominal cavity. The right Fallopian tube and ovarian ligament were ligated, cut and dropped into the abdomnal cavity, in the same manner as the pedicle; as they were found firmly adherent by inflammation to the tumor, or rather the ovary was apparently absorbed, as it was not found. The incision was closed by five silver wire sutures including the peritoneum, and three superficial sutures of carbolized silk. The sutures were removed on the tenth day, and the whole abdomen covered with resin adhesive plaster in such manner as to keep the wound united and support the abdominal walls.

This operation was also conducted according to Lister' antiseptic method.

The wound healed by first intention, and the recovery of the patient progressed with almost no disturbance of the system.

*Case III.* Miss H. F., a teacher, aged 24, born in Canada of Scotch parents, was admitted into the Hospital for Women and Children Jan. 28. 1879.

Health always good. Menstruation began at age of fifteen; never painful, and always flowed freely three or four days.

Came to the West about eighteen months ago. Gradually "increased in flesh" after leaving Canada. First noticed abdominal enlargement July, 1878. In August, her stomach became so irritable that she could eat almost nothing until the past month. Menses, scanty and painful in September, 1878, ceased entirely in November. The enlargement increased rapidly after the menses ceased. During the last period (in which she should have menstruated) before the operation, which occurred just after her entering the hospital, her size increased, so rapidly as to be noticed by every one who saw her daily, and considerable œdema of the feet came on.

During the summer the urine was dark and contained a pink sediment. A few weeks ago it had a "milky look." At present it is free, with sediment of urates and phosphates.

During the past month her health has been good, except a pain over the left portion of her abdomen and across the lower part of her back.

On examination by Dr. Thompson the fundus of the uterus was found pressed forward between the pubes and the tumor, posteriorly. Fluctuation was detected in the abdomen. Measurement, 97 Ctm. at the umbilicus.

On Feb. 5, at 2.15 p. m., the patient was anæsthetized by Dr. Bottsford, with the intention of concluding the diagnosis by aspiration, but on examination by Drs. Bogue and Thompson the case seemed so clearly one of ovarian tumor, that it was decided to proceed in the usual manner for the removal of the tumor. Physicians present were Drs. Thompson, Bogue, Bartlett, Groesbeck, Bottsford, Gaston, Barlow and Ballard; besides Miss Akers and others.

Dr. Thompson, assisted by Drs. Bogue, Bartlett and Gaston, performed the operation under the carbolized spray. An incision was made in the median line, which extended from about 2 Ctm. below the umbilicus downward about 10 Ctm. When the sac was reached, Spencer Wells' trocar, with tubing attached, was thrust into it. The operator then tried to introduce her hand to break up the adhesions; but finding the incision too small to work to advantage, enlarged it about 1½ Ctm. Adhesions were then found over the superior portion of the tumor, which were severed; and the sac was then removed from the abdominal cavity. The pedicle was pierced near the uterus by a large needle armed with a double silk braid (carbolized), and the ligatures tied upon opposite sides, dividing it into two parts. The pedicle was then cut, leaving a short stump of about 1 Ctm. in length, and dropped back into the abdominal cavity. On examination of the right ovary, it was found to contain one or more cysts, and was removed with the outer portion of the Fallopian tube. The hæmorrhage was quite free, but no vessels were ligated. The cavity was carefully sponged out to cleanse it of the blood which had poured into it. Evidence of considerable inflammation existed in the thickened, rough and dark red peritoneum.

The incision was closed with one superficial and eight deep sutures, all of silver wire; but in twisting the wires, the second and third from the lower end of the wound broke, and superficial wire sutures were put in their places.*

---

*This wire was supposed to have been affected by lying in carbolized water for an hour at a time at several different times.

Lister's dressings and flannel bandage were applied, and the patient put to bed at 3:30, P. M. Pulse 108.

Fluid from the cyst measured 14 litres, and weighed 15 kilo. The cyst weighed 2 kilo.

At 5:30, P. M., when the patient awoke from the effects of the anæsthetic, she was given 0.15 of quinine and 0.02 of morphia. 7 P. M., pulse 120; 9 P. M., pulse 132, temp. 39°.

Sixth, 5 A. M. Vomited; could only tolerate milk diet; 3 P. M. vomited again. Pulse, 116.

Seventh. Nausea continued until this morning, and could take no food.

Eighth, 7 A. M. Temp. dropped to 37.3°; pulse, 88.

Ninth, 8 P. M. Temp. 38.4°; pulse, 102. Both gradually dropped to the *normal standard* in the early part of the succeeding day.

Tenth. Removed five sutures.

Eleventh. The remaining sutures were removed and adhesive straps applied.

Fourteenth. Patient removed to another bed. In two weeks from the time of the operation she was apparently well, but weak; at this date reports herself well.

In the last two cases there was not even a drop of pus in or about the incisions or sutures. It was Dr. Thompson's opinion that the nausea and vomiting in the last case were caused by the morphine, which was changed to tr. opii then to tr. opii et camph.

